# ISMB/ECCB 2023 organization benefited from the strengths of the French bioinformatics community

**DOI:** 10.1093/bioadv/vbae040

**Published:** 2024-05-03

**Authors:** Anna-Sophie Fiston-Lavier, Sandra Dérozier, Guy Perrière, Marie-France Sagot

**Affiliations:** ISEM, Université Montpellier, CNRS, IRD, Montpellier, France; Institut Universitaire de France (IUF), France; Université Paris-Saclay, INRAE, MaIAGE, Jouy-en-Josas 78350, France; Univ Lyon et UMR CNRS 5558, France; UAR CNRS 3601 IFB-Core, France; Univ Lyon et UMR CNRS 5558, France; Inria, CNRS and Univ. of Lyon 1, France

The International Society of Computational Biology—ISCB (https://www.iscb.org) was founded in 1997 as a non-profit organization dedicated to all aspects of the development of our understanding of living organisms using computational and mathematical methods. ISMB is the annual International Conference on Intelligent Systems for Molecular Biology, which is the flagship meeting of ISCB and was established in 1993. ECCB is the European Conference on Computational Biology, which was first held in 2002. In 2023, the French bioinformatics community collaborated with ISCB to organize a joint international conference in France, with great success. Here, we first describe the strengths of the French bioinformatics community and then how they contributed to the success of ISMB/ECCB 2023.

France is fortunate to have a close-knit, dynamic community in the field of bioinformatics in general, with a national conference devoted to it every year since 2000. This conference is called JOBIM, which stands for “Journées Ouvertes en Biologie, Informatique et Mathématiques” (meaning Open days in Biology, Computer Science and Mathematics; Fig. 1). The conference is quite large, bringing together between three and five hundred participants each year. The JOBIM conference is about science, fundamental and applied, but also about supporting cooperation between researchers involved in this science and, nurturing friendship. In all JOBIM conferences, since 2000, we have keynote presentations, talks and poster sessions, but also dedicated moments to relax and chat amongst ourselves. The French bioinformatic groups contribute to making JOBIM a very attractive event for young bioinformaticians by offering a relaxed atmosphere to meet other scientists. To ensure that such a conference would continue to be available to the community, the “Société Française de BioInformatique” (SFBI) (https://www.sfbi.fr/) was founded in 2005. The main aim of this society is to bring together the French bioinformatic community by organizing an annual conference. Since its inception, SFBI has expanded to take on other missions, for the benefit of the community. SFBI promotes interdisciplinary research in France and brings together the French-speaking community for scientific meetings and exchanges. SFBI also promotes training by providing a catalog of diploma courses and long-term training in bioinformatics, encourages the participation and integration of young bioinformaticians into scientific events by offering travel fellowships and awards, and contributes to the visibility of bioinformatics in France by supporting science and outreach bioinformatic events. Since 2021, SFBI has been a founding and active member of the College of French Academic Societies (https://societes-savantes.fr) to defend positions on more general issues concerning research in our society (Fig. 1).

**Figure 1.**
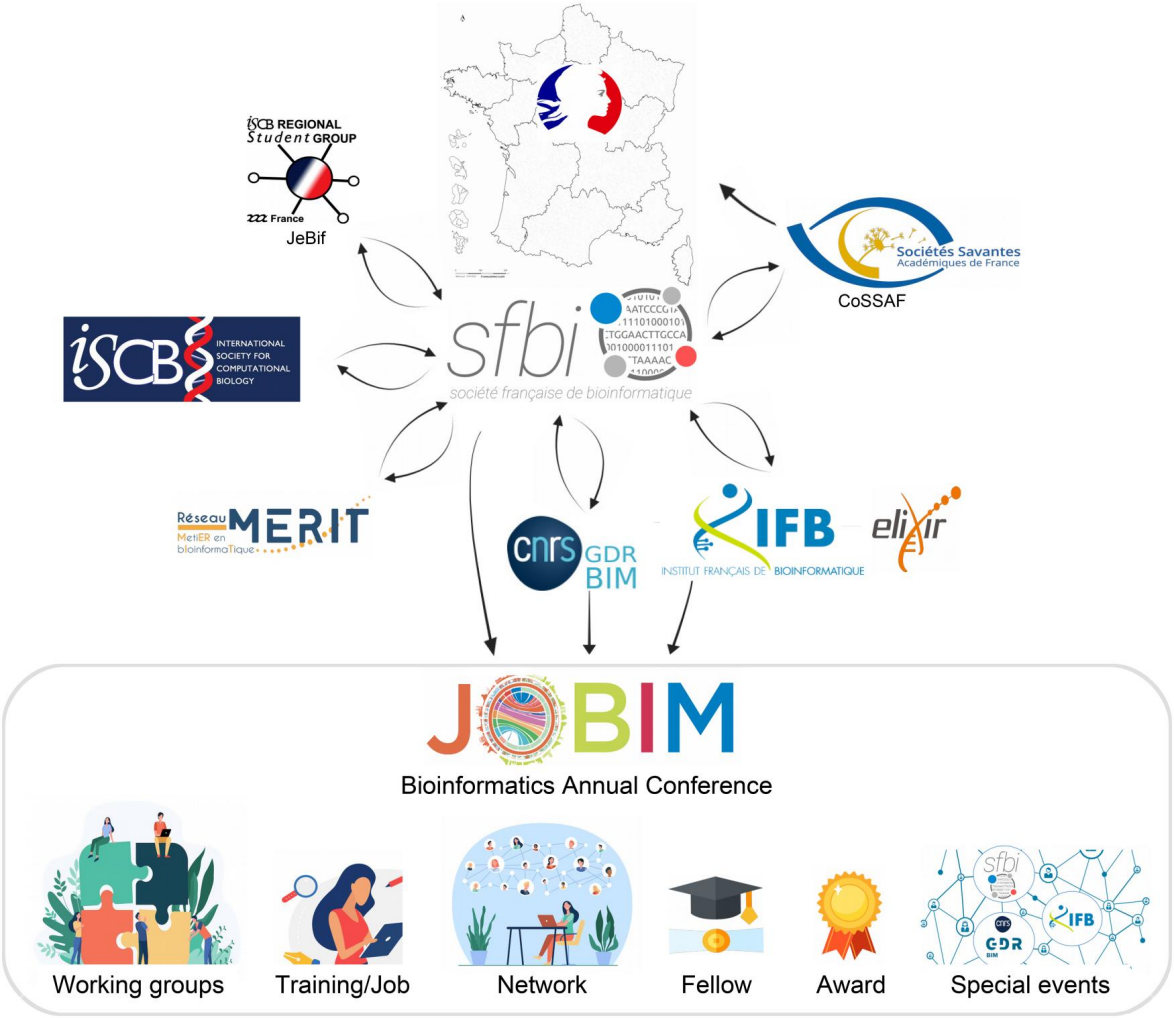
The French bioinformatics community. In France, several groups exist to support the bioinformatics community. These groups interact with the SFBI and have missions complementary to those of the SFBI. Through Cossaf, the SFBI can communicate specific requests or needs from the community to the research ministries. Altogether, they animate the community by organizing JOBIM (the annual bioinformatics conference), by launching working groups on specific topics, by providing several resources such as catalogs of training/jobs as well as the list of bioinformatics laboratories or teams, by offering fellowships and special awards for young bioinformaticians, and by supporting other bioinformatics events such as the special session “Bioinformatics in France” of the ISMB/ECCB 2023.

Besides the SFBI, other significant groups also exist to support French bioinformatics, with missions that complement those of SFBI and work with the SFBI (Fig. 1). The French Bioinformatics Institute (https://www.france-bioinformatique.fr/) is a network of facilities as well as a research unit forming the French European infrastructure in bioinformatics (https://www.france-bioinformatique.fr/en/elixir-fr/). As a national service infrastructure, the mission of IFB is to offer the communities of life sciences and computational biology, both academic and private, access to resources critical to their research, support for research projects, and the opportunity to participate in ambitious projects at the national and international level. In collaboration with SFBI, it provides a catalog of training in bioinformatics in France to help biologists, computer scientists, bioinformaticians, mathematicians and other scientists to gain new expertise. IFB’s active engagement at the cutting edge of developments in bioinformatics ensures the positioning of French research as an essential actor in the field, particularly in meeting integrative bioinformatic challenges. The Molecular Bioinformatics Group, also called GDR-BiM, belongs to one of France's largest public scientific research organizations called “*Centre National de la Recherche Scientifique”* (CNRS) and is a network dedicated to promoting Bioinformatics Research (https://www.gdr-bim.cnrs.fr/). The GDR-BiM has a number of thematic working groups with forums and supports the organization of scientific events. With IFB, the GDR-BiM is a long-standing partner in the organization of JOBIM. JeBIF for “Jeunes Bioinformaticiens de France',” a group dedicated to young bioinformaticians related to the Student Council of ISCB, is the Regional Student Group in France. JeBiF aims to create an active community to stimulate local and national synergies by encouraging the establishment of collaborative projects between public research and private companies, as well as partnerships between its members to develop engaging scientific activities. It promotes the French community in bioinformatics to public institutions and private entities at both national and international levels, making this discipline known to the general public. In collaboration with the SFBI, JeBIF maintains a catalog of the master programs in bioinformatics in France. Finally, to complete the picture, a recently established network of bioinformatic engineers (2023) (https://merit.cnrs.fr/) is currently being set up to promote the organization of working groups at a regional or national level, to propose and organize training courses and to work towards recognition of the bioinformatics profession (Fig. 1).

The bioinformatics community is also well-structured internationally, thanks to ISCB which organizes the ISMB conference every year and the ISMB/ECCB conference in Europe every two years by joining forces with ECCB. The ISMB/ECCB 2023 conference was planned in France. The ISCB organizing committee invited the SFBI to join the committee board of the ISMB/ECCB 2023 conference. Thanks to our collective expertise, we created a synergy to organize the ISMB/ECCB 2023 conference, the first international conference on bioinformatics and computational biology that included also the national society in those areas of research. We ended up with 2 485 confirmed attendees worldwide, with 2 067 on-site and 418 online (Fig. 2). Among the participants, 242 were from France, thus making it the third most represented country in attendance (Fig. 2). For the large majority of the countries, including France, most attendees were students and professionals (Academic; Non-profit; Government; or Corporate). There were few registrations of postdocs and of other participants, such as employees of private companies (Fig. 2).

**Figure 2.**
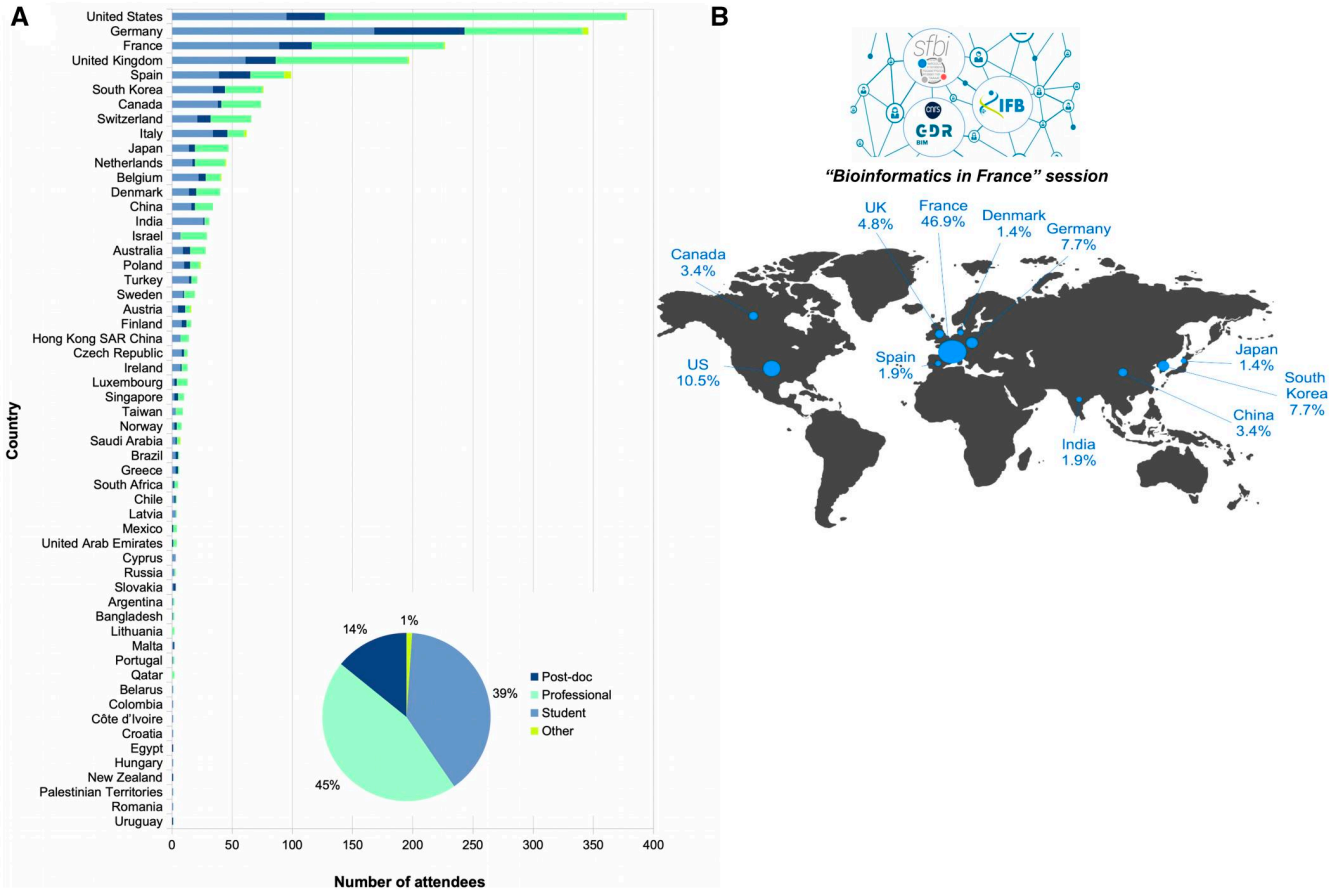
(A) Distribution of the 2067 confirmed on-site attendees of the ISMB/ECCB 2023 conference, among countries based on their status (Post-Doc, Professional, Student, and Other). (B) Geographic distribution of the 209 attendees of the special session “Bioinformatics in France.” Only the countries with more than 1% of attendees are represented.

In addition to its role on the conference organizing committee, SFBI's contribution was manifold. This involved keeping the committee informed about the local situation in France, such as the local rules to be adopted during the pandemic, or the disruptions to be expected due to strikes. SFBI was also able to act as an interpreter and deal with some service providers before and during the conference. Compared to ISMB/ECCB 2019 at Basel in Switzerland, with only 72 French participants mainly represented by professionals, for this new edition the French community was well represented. The involvement of SFBI and the communication through its network strongly motivated the community members to take part in this new edition, and more specifically the students and other young bioinformaticians, by providing registration discount codes.

The SFBI also notably played a crucial role in organizing for the first time a Special Session “Bioinformatics in France” to present the French Bioinformatics community to international colleagues (https://www.sfbi.fr/blog/article/ismbeccb2023-special-session-bioinformatics-infrance). Out of the 2 067 on-site confirmed attendees, 209 registered for the “Bioinformatics in France” session. Half of the attendees who registered for this session (53.1%) came from outside France with 21.1% from Europe, 14.4% from the American continent, 17.2% from the Asian continent and 0.5% from Oceania (Fig. 2). This special session was organized in three parts. The first included talks by the main French groups in bioinformatics, namely SFBI, GDR-BIM, and IFB-Elixir. The idea was to give an overview of the French bioinformatic community.

The second part was dedicated to the scientific presentations of two dynamic members of the French community. Flora Jay, a researcher at the *Laboratoire Interdisciplinaire des Sciences du Numérique*/interdisciplinary computer science laboratory (LISN) of the University Paris-Saclay, presented her work entitled “*Design and Application of deep neural networks for population genetics”.* Yann Ponty, a research director, and head of the Algorithms and Models for Integrative Biology team of the Computer Science laboratory of *École Polytechnique*, then gave a presentation entitled *RNA Bioinformatics: Still combinatorial in 2023?*. To offer the opportunity for bioinformaticians to present their work, we also selected six high-quality abstracts from students for short talks (https://www.sfbi.fr/blog/article/ismbeccb2023-specialsession-bioinformatics-in-france). Finally, all the session attendees and the speakers were invited to close the day with a social event.

The “Bioinformatics in France” special session was the first one of its kind dedicated to the research being conducted in the country hosting ISMB and ECCB, and to the national community involved in such research (https://www.sfbi.fr/blog/article/ismbeccb2023-specialsession-bioinformatics-in-france). We do deeply thank both conference boards and ISCB for having set up such a collaborative effort. The involvement of the local community in the organization of the ISMB/ECCB 2023 conference was a real benefit for both junior bioinformaticians who were encouraged to take part in an international conference and the conference itself, which gained local information to help its organization. We hope this will strongly inspire other countries to organize their community in order to encourage such Special Sessions to take place in future host countries.

## Data Availability

There are no new data associated with this article.

